# Supplementary material for: High Prevalence of the Lung Ultrasound Interstitial Syndrome in Systemic Sclerosis Patients with Normal HRCT and Lung Function—A Pilot Study
Source: J Clin Med. 2024 May 14;13(10):2885. doi: 10.3390/jcm13102885 (PMC11121911; doi:10.3390/jcm13102885)
Supplement: Supplementary file 1 [file jcm-13-02885-s001.zip › jcm-2942415-supplementary.pdf]

Supplementary Materials

	Anatomical lines	Right	Left
Anterosuperior	Para-sternal	2nd LIS	2nd LIS
	Mid-clavicular	4th LIS	4th LIS
	Anterior axillary	4th LIS	4th LIS
posterobasal	Mid axillary	4th LIS	4th LIS
	Posterior axillary	8th LIS	8th LIS
	Sub scapular	8th LIS	8th LIS
	Para-vertebral	8th LIS	8th LIS

Abbreviations LIS : Lung intercostal space

Supplementary Figure S1. List of the 14 thoracic areas explored.

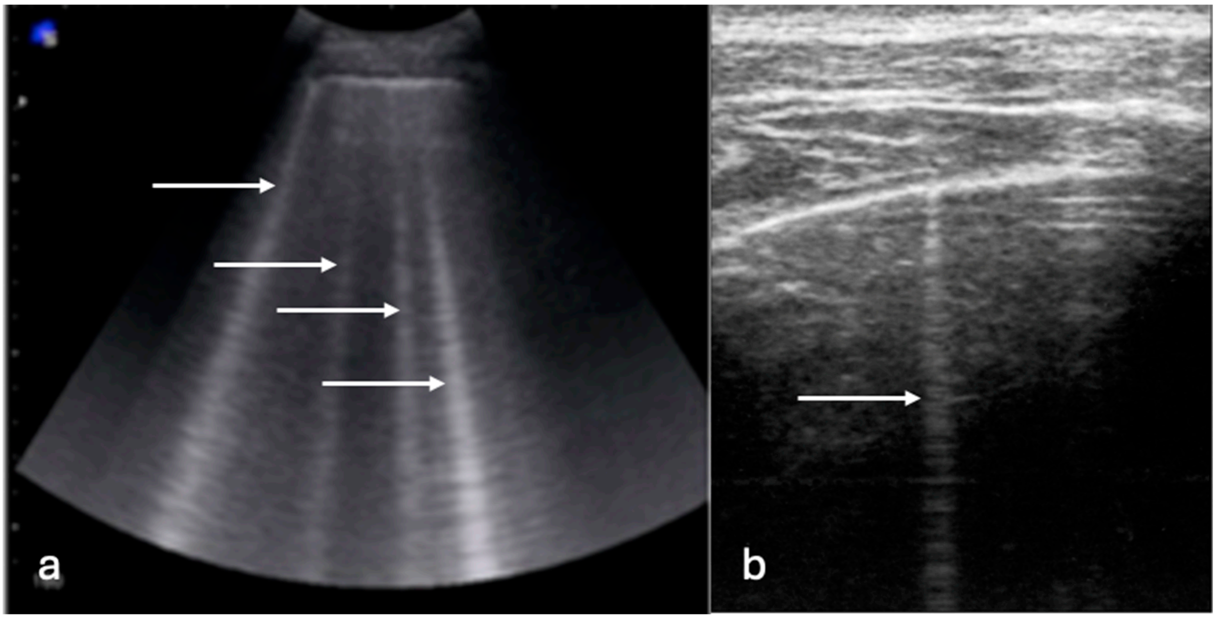

Supplementary Figure S2: The arrows show the number of B-lines. (**a**): 4 B-lines, (**b**): 1 B-line

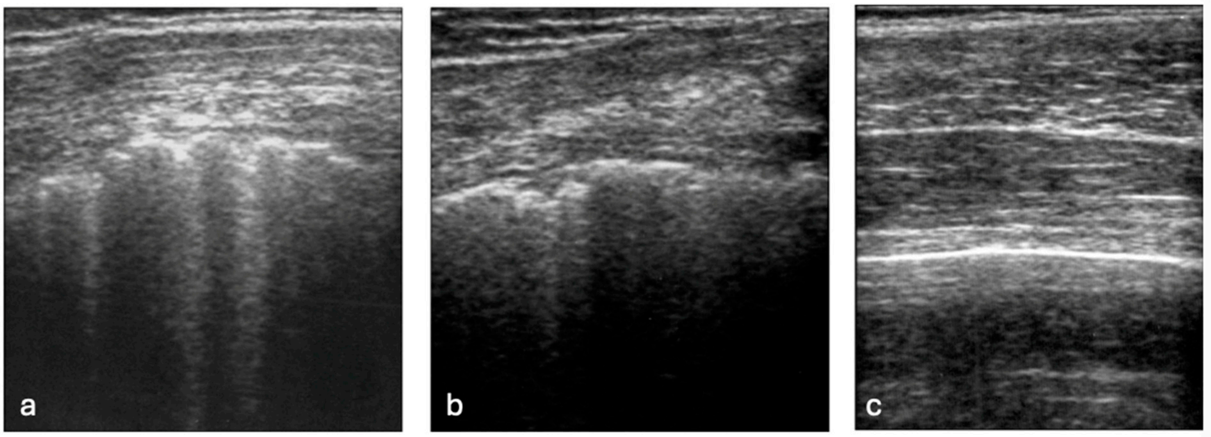

Supplementary Figure S3: Irregularity of pleural line. (**a**,**b**): presence of an irregularity, (**c**) absence of irregularity

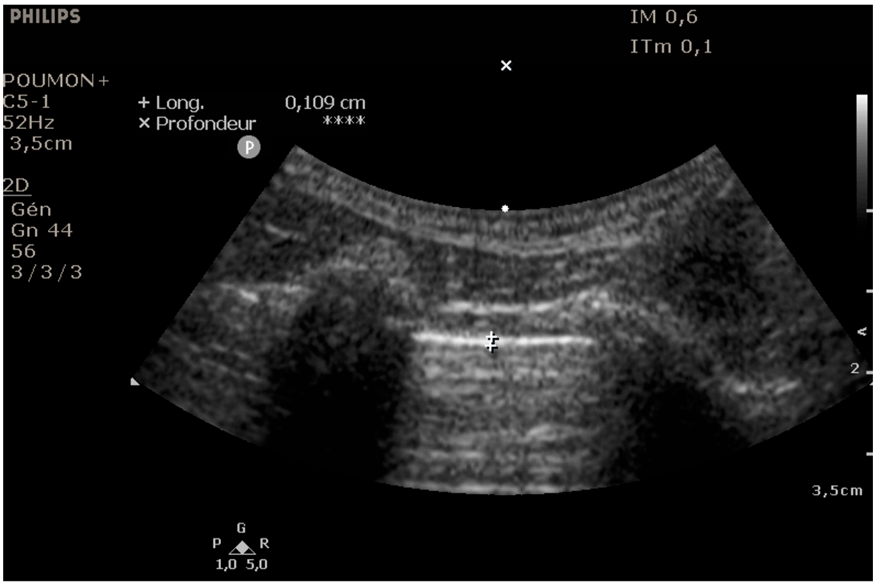

Supplementary Figure S4: Pleural line measurement. Here: 1.09 mm.

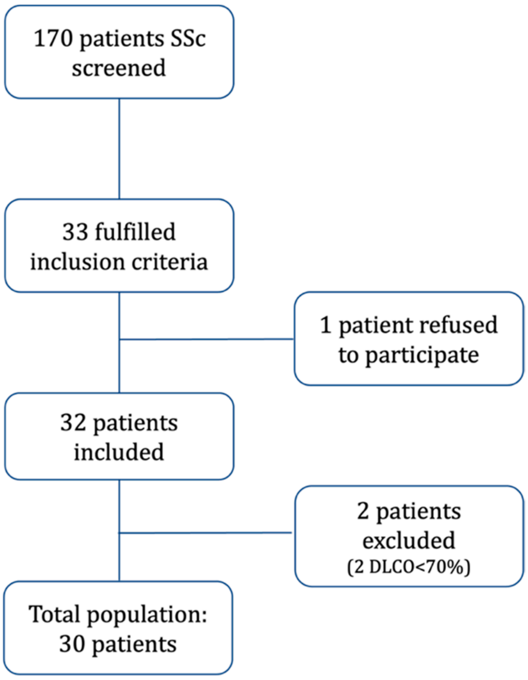

Supplementary Figure S5. Flowchart of the study. Abbreviations: DLCO: diffusing capacity for carbon monoxide; ILD: interstitial lung disease; SSc: systemic sclerosis.

Supplementary Table S1. Localisation of B lines, pleural line irregularity, and pleural line thickness ≥3 mm among the 14 thoracic areas.

		Para-sternal	Mid-clavicular	Anterior axillary	Mid-axillary	Posterior axillary	sub scapular	Para-vertebral
**B lines**	R	4	5	7	6	11	6	4
	L	3	3	5	6	6	6	11
	Sum	7	8	12	12	17	12	15
	%	8.43	9.64	14.46	14.46	20.48	14.46	18.07
**Irregularity Pleural line**	R	5	4	0	2	6	2	7
	L	3	1	0	4	6	3	5
	Sum	8	5	0	6	12	5	12
	%	16.67	10.42	0	12.50	25.00	10.42	25.00
**Pleural line thickness**	R	1	0	0	0	1	0	0
	L	0	0	0	0	1	0	1
	Sum	1	0	0	0	2	0	1
	%	25.00	0	0	0	50.00	0	25.00

Abbreviations: R: right; L: left. % represent the repartition of ultrasound signs (B lines, irregularity and line thickness) over the all 14 thoracic areas. Bold represents a percentage > 20%.
